# Major Adverse Cardiovascular Events and Cardiac Dysfunction in Patients With Acute Leukemia: A Prospective Study

**DOI:** 10.1016/j.jaccao.2025.11.004

**Published:** 2026-01-16

**Authors:** Yu Kang, Benedicte Lefebvre, Amanda Smith, Daniel Koropeckyj-Cox, Priya Brahmbhatt, Bonnie Ky, Joseph Carver, Shannon McCurdy, Selina Luger, Marielle Scherrer-Crosbie

**Affiliations:** a Division of Cardiovascular Disease, Department of Medicine, Hospital of the University of Pennsylvania, Philadelphia, Pennsylvania, USA; b Thalheimer Center for Cardio-Oncology, Perelman School of Medicine at the University of Pennsylvania, Philadelphia, Pennsylvania, USA; c Division of Hematology and Oncology Disease, Department of Medicine, Hospital of the University of Pennsylvania, Philadelphia, Pennsylvania, USA

**Keywords:** acute leukemia, anthracyclines, cardiac dysfunction, major adverse cardiovascular events

Acute leukemia is life threatening, with about 28,000 new cases and 13,000 deaths in the United States in 2024.^1^ Although the incidence is increasing and mortality has declined, few trials address comorbidities and treatment side effects,^2^ underscoring critical gaps in both knowledge and care for this population. The prevalence of symptomatic heart failure (HF) in acute leukemia patients treated with anthracyclines is higher than in patients with other cancers treated with anthracyclines.^3^ Acute coronary syndrome, ischemic stroke, thromboembolism, and cardiovascular-related death have also been noted in patients with acute myeloid leukemia (AML) treated with anthracycline-based chemotherapy.^4^ The aim of this prospective study was to characterize major adverse cardiac events (MACE) and cardiac function in acute leukemia patients.

Adult patients with newly diagnosed acute leukemia were prospectively enrolled, and visits with echocardiograms were scheduled prior to and 14 days, 1 to 3, 6, and 12 months after chemotherapy. The primary endpoint was symptomatic MACE, including symptomatic HF, acute coronary syndrome, stroke, new-onset atrial fibrillation, and cardiac death. The secondary endpoint was cardiac dysfunction (CD), defined as symptomatic left ventricular ejection fraction (LVEF) decline of ≥5% to <55% or asymptomatic decline of ≥10% to <55%. The cumulative incidence rates of both MACE and CD were estimated, with noncardiac death as a competing event. Time-dependent Cox proportional hazards model (MACE or CD) or Kaplan-Meier survival analysis (subtypes of leukemia) was used to analyze the associations between clinical parameters and all-cause mortality. Fine and Gray’s subdistribution hazard regression analysis assessed the associations between clinical parameters and either MACE or CD, with noncardiac death as a competing event.

Seventy-four patients (47 men [63.5%]; age range: 22-83 years; mean age 54 ± 15 years) (Table 1) were included, with a median follow-up duration of 341 days (range: 14-1,084 days; Q1-Q3: 187-451 days). Fifty-seven patients (77.0%) received anthracyclines (doxorubicin equivalent dose 191 mg/m^2^; Q1-Q3: 149-270 mg/m^2^; range: 50-404 mg/m^2^). Twenty patients (27.0%) died of noncardiac causes, with a median time to death of 326 days (Q1-Q3: 169-529 days), including 8 of infection, 7 of disease progression, 2 of pulmonary embolism, and 3 of unknown or unwitnessed events.

**Table 1:** Clinical Characteristics of the Study Cohort

	All (N = 74)	No MACE (n = 65)	MACE (n = 9)	No CD (n = 58)	CD (n = 16)
Demographics					
Age, y	54 ± 15	53 ± 15	65 ± 9	54 ± 16	55 ± 11
Male	47 (63.5)	22 (33.8)	6 (66.7)	38 (65.5)	3 (18.8)
BMI, kg/m^2^	30.0 ± 7.5	30 ± 8	31 ± 4	29.7 ± 8.1	31.1 ± 5.0
Follow-up, d	341 (187-451)	342 (209-457)	272 (89-375)	353 (240-447)	236 (117-565)
Cardiovascular history					
Hypercholesterolemia	23 (31.1)	18 (27.7)	5 (55.6)	19 (32.8)	4 (25.0)
Hypertension	25 (33.8)	23 (35.4)	3 (33.3)	20 (34.5)	6 (37.5)
Diabetes	13 (17.6)	11 (16.9)	2 (22.2)	11 (19.0)	2 (12.5)
Obesity	34 (45.9)	28 (43.1)	6 (66.7)	24 (41.4)	10 (62.5)
Smoking	25 (33.8)	21 (32.3)	2 (22.2)	21 (36.2)	4 (25.0)
≥2 CV risk factors^a^	28 (37.8)	24 (36.9)	4 (44.4)	21 (36.2)	7 (43.8)
Coronary heart disease	5 (6.8)	5 (7.7)	0 (0)	3 (5.2)	2 (12.5)
Atrial fibrillation/flutter	4 (5.4)	4 (6.2)	0 (0)	3 (5.2)	1 (6.3)
Chronic kidney disease	4 (5.4)	4 (6.2)	0 (0)	2 (3.4)	2 (12.5)
Cerebrovascular disease	2 (2.7)	1 (1.5)	1 (11.1)	2 (3.4)	0 (0.0)
Pre-existing CV disease^b^	12 (16.2)	11 (16.9)	1 (11.1)	8 (13.8)	4 (25.0)
Prior malignancy	21 (28.4)	16 (24.6)	5 (55.6)	15 (25.9)	6 (37.5)
Cardiovascular medicine (prior to leukemia diagnosis)					
Beta-blockers	11 (14.9)	9 (13.8)	3 (33.3)	8 (13.8)	4 (25.0)
ACEIs/ARBs	12 (16.2)	12 (18.5)	0 (0)	9 (15.5)	4 (25.0)
Aspirin	8 (10.8)	6 (9.2)	2 (22.2)	8 (13.8)	0 (0.0)
Anticoagulant agents	3 (4.1)	3 (4.6)	0 (0)	1 (1.7)	2 (12.5)
Statins	22 (29.7)	18 (27.7)	4 (44.4)	17 (29.3)	5 (31.3)
Cancer subtype and cancer-related treatment					
AML	49 (66.2)	41 (63.1)	8 (88.9)	37 (63.8)	12 (75.0)
Anthracycline	57 (77.0)	51 (78.5)	6 (66.7)	41 (70.7)	16 (100.0)
Anthracycline dose, mg/m^2^	191 (149-270)	180 (149-270)	224 (191-285)	180 (149-270)	232 (184-272)
All-trans retinoid acid/arsenic trioxide	4 (5.4)	4 (6.2)	0 (0)	4 (6.9)	0 (0)
Venetoclax/azacitidine	11 (14.9)	9 (13.8)	2 (22.2)	11 (19.0)	0 (0)
Other treatment	2 (2.7)	1 (1.5)	1 (11.1)	2 (3.4)	0 (0)
Allogeneic stem cell transplantation	36 (48.6)	34 (52.3)	2 (22.2)	28 (48.3)	8 (50.0)
Time to transplantation, d	161 (105-556)	164 (130-217)	130 (121-140)	164 (139-218)	139 (115-183)
Outcomes					
All-cause death	23	17	6	16	7
Time to death, d	272 (151-386)	243 (155-457)	305 (120-536)	327 (233-493)	144 (68-337)

Values are mean ± SD, n (%), or median (Q1-Q3). Percentages are row-wise percentages.

ACEI = angiotensin-converting enzyme inhibitor; AML = acute myeloid leukemia; ARB = angiotensin receptor blocker; BMI = body mass index; CD = cardiac dysfunction; CV = cardiovascular; MACE = major adverse cardiac event.

a CV risk factors included obesity, hypertension, hypercholesterolemia, and diabetes.

b CV diseases included coronary artery disease, atrial fibrillation or flutter, cerebrovascular disease, and chronic kidney disease.

There were 13 episodes of symptomatic MACE in 9 patients. MACE included acute coronary syndromes (n = 3), new-onset atrial fibrillation (n = 1), and acute ischemic strokes (n = 5). Eight patients had arterial thrombotic MACE (3 acute coronary syndromes and 5 ischemic strokes). The median time to first MACE was 128 days (range: 3-353; Q1-Q3: 26-198 days). Patients with MACE were older; no other differences were noted in terms of cardiovascular risks and history, type of leukemia, or cancer treatment, including allogeneic stem cell transplantation. In the whole cohort, the 6-month and 12-month cumulative incidence of MACE was 8.5% (95% CI: 2.2%-14.8%) and 12.2% (95% CI: 8.5%-15.9%), respectively. Three patients (33.3% of patients with MACE) died within 1 week following ischemic stroke. Patients with MACE had a higher all-cause death rate; 6-month and 12-month cumulative incidence rates were 25.4% (95% CI: 23.2%-27.6%) and 69.8% (95% CI: 67.4%-72.2%) vs 10.4% (95% CI: 5.3%-16.0%) and 23.9% (95% CI: 18.3%-29.5%) in patients without MACE (time-dependent Cox model, *P* = 0.013).

Sixteen of 70 patients (excluding 4 with reduced baseline LVEFs) developed CD; 1 experienced symptomatic HF. In patients with CD, the lowest LVEF was 47% ± 5%, with a median time to nadir of 68 days (range: 22-186 days; Q1-Q3: 50-159 days). In the whole cohort, the 6- and 12-month cumulative incidence of CD was 23.2% (95% CI: 13.1%-33.2%) and 23.2% (95% CI: 13.1%-33.2%), respectively. Twelve of these patients had AML, and 4 had acute lymphoblastic leukemia (ALL). All received anthracyclines (median doxorubicin equivalent dose 232 mg/m^2^; Q1-Q3: 184-272 mg/m^2^; range: 100-397 mg/m^2^), and cardioprotective medications (β-blockers in 10 patients, angiotensin-converting enzyme inhibitors or angiotensin receptor blockers in 9 patients, and mineralocorticoid receptor antagonists in 2 patients). The median time to initiating new cardioprotective therapy after LVEF decrease was 30 days (Q1-Q3: 27-42 days). In 7 patients, LVEF recovered to ≥55%, and in another patient, LVEF recovered to between 50% and 54%, with a median recovery time of 207 days (range: 56-471 days; Q1-Q3: 100-353 days). Nine of 70 patients (excluding the 4 patients with low baseline LVEFs) experienced greater reductions of LVEF to <50%, all with AML and anthracycline exposure (median doxorubicin equivalent dose 188 mg/m^2^; Q1-Q3: 149-270 mg/m^2^; range: 180-397 mg/m^2^). Each 10 mg/m^2^ increase in doxorubicin equivalent dose was associated with an approximately 6% higher risk for CD (subdistribution HR: 1.06; 95% CI: 1.02-1.10; *P* = 0.002).

Seven patients with CD died; causes included pulmonary embolism (n = 2), infections (n = 2), cancer progression (n = 1), and unwitnessed events (n = 2). Patients experiencing CD had a higher all-cause death rate, with 6-month and 12-month rates of 35.2% (95% CI: 31.6%-38.8%) and 45.3% (95% CI: 41.0%-49.6%) vs 7.5% (95% CI: 1.3%-13.1%) and 27.6% (95% CI: 21.0%-33.2%) without CD (time-dependent Cox model, *P* = 0.043). After adjusting for age, CD remained associated with higher all-cause mortality (HR: 3.1; 95% CI: 1.1%-8.5%; *P* = 0.028).

Among 37 patients with allogeneic hematopoietic stem cell transplantation, 2 had MACE (1 stroke and 1 acute coronary syndrome), 8 had CD (median time to CD 154 days; Q1-Q3: 36-174 days), including 3 post-transplantation (at 4, 52, and 72 days), and 5 deaths.

Forty-nine patients had AML (66.2%), 21 had ALL (28.4%), and 4 acute promyelocytic leukemia (5.4%). In comparison with patients with ALL patients, AML patients were older, received higher anthracycline doses, and had a higher death rate. The 6-month and 12-month cumulative incidences of all-cause death were 17.4% (95% CI: 17.3%-17.5%) and 43.0% (95% CI: 42.8%-43.2%) in patients with AML and 5.3% (95% CI: 5.2%-5.4%) and 11.2% (95% CI: 11.0%-11.4%) in ALL patients, respectively (log-rank *P* = 0.022). The 6-month and 12-month cumulative incidences of MACE were 10.5% (95% CI: 3.8%-21.1%) and 16.4% (95% CI: 6.9%-29.6%) in patients with AML and 4.8% (95% CI: 0.3%-20.2%) and 4.8% (95% CI: 0.3%-20.2%) in those with ALL, respectively (cumulative incidence function, *P* = 0.36). The 6-month and 12-month cumulative incidences of CD were 23.6% (95% CI: 12.5%-36.6%) and 25.8% (95% CI: 14.2%-39.1%) in patients with AML and 19.6% (95% CI: 5.8%-39.2%) and 19.6% (95% CI: 5.8%-39.2%) in those with ALL, respectively (cumulative incidence function, *P* = 0.63). In 37 patients (mean age 54 ± 12 years) with AML receiving anthracycline-based chemotherapy (median doxorubicin equivalent dose 268 mg/m^2^; Q1-Q3: 180-273 mg/m^2^), 12 patients experienced CD. Of note, the 9 patients with more marked LVEF reductions (≥10% to ≤50%) all had AML and were treated with anthracyclines.

In summary, in this prospective study, approximately 12% of patients with acute leukemia developed symptomatic MACE during a 12-month follow-up. The majority of MACE were acute arterial thrombotic events, affecting 11% of patients and occurring at a median time of 4 months. CD occurred in 23% of patients overall over 12 months; a more marked decrease of LVEF to ≤50% was present only in patients with AML treated with anthracyclines. Because of the limited sample size, we were unable to further explore other potential covariates that might influence overall mortality. Nevertheless, the observed associations between MACE or CD and higher mortality rate highlight their prognostic importance. Further studies are needed to validate our results with larger, more diverse cohorts.

## Funding Support and Author Disclosures

This study was funded by grant R01 HL130539 (to Dr Scherrer-Crosbie). The authors have reported that they have no relationships relevant to the contents of this paper to disclose.

## References

[1] Siegel R.L., Giaquinto A.N., Jemal A. (2024). Cancer statistics, 2024. CA Cancer J Clin.

[2] Crossnohere N.L., Richardson D.R., Reinhart C. (2019). Side effects from acute myeloid leukemia treatment: results from a national survey. Curr Med Res Opin.

[3] Kang Y., Assuncao B.L., Denduluri S. (2019). Symptomatic heart failure in acute leukemia patients treated with anthracyclines. JACC CardioOncol.

[4] Calvillo-Arguelles O., Schoffel A., Capo-Chichi J.M. (2022). Cardiovascular disease among patients with AML and CHIP-related mutations. JACC CardioOncol.

